# Confirmatory Factor Analysis of the French Version of the Anticipatory and Consummatory Interpersonal Pleasure Scale

**DOI:** 10.3389/fpsyg.2017.01296

**Published:** 2017-07-28

**Authors:** Joséphine Chaix, Philippe Golay, Caroline Fankhauser, Alexandra Nguyen, Diane C. Gooding, Jérôme Favrod

**Affiliations:** ^1^School of Nursing Sciences La Source, University of Applied Sciences and Arts of Western Switzerland Lausanne, Switzerland; ^2^Community Psychiatry Service, Department of Psychiatry, University Hospital Center Lausanne, Switzerland; ^3^Department of Psychology, University of Wisconsin-Madison, Madison WI, United States

**Keywords:** social anhedonia, bonding, confirmatory factor analysis, ACIPS, validation, self–report scale

## Abstract

The Anticipatory and Consummatory Interpersonal Pleasure Scale (ACIPS), a measure specifically designed to assess hedonic capacity for social and interpersonal pleasure, was used to evaluate the presence of social anhedonia in patients as well as the general population. The first goal of this study was to validate the structure of the French version of the ACIPS. The second objective was to verify whether a one, two or three factor solution is most appropriate for the ACIPS scale. The French version of the ACIPS was tested on 263 French-speaking pre-graduate students or professional volunteers. For the confirmatory factor analysis, data were treated as categorical ordinal and all the models were estimated using a robust weighted least squares estimator with adjustments for the mean and variance. Three models were estimated. A one-factor model representing a general undifferentiated “pleasure” latent construct was first tested on the 17 ACIPS items. A two-factor model distinguishing anticipatory-pleasure and consummatory-pleasure was tested next. Finally, a three-factor model including subdomains of intimate social interactions, group social interactions, and social bonding was tested. The one and two-factor models showed a somewhat poor fit to the data. However, the goodness of fit of the three factor model was adequate. These results suggest that individuals who enjoyed interaction in one of these three subdomains were more likely to enjoy doing so in the two other domains. However, on the basis of the comparison between the one and three factor models, these three types of interactions may not be considered as indistinguishable. Rather, they represent distinct and theoretically meaningful dimensions. These results show the French version of the ACIPS is a useful and valid scale to measure the capacity of savoring different kinds of social relationships.

## Introduction

Anhedonia is defined as the diminished capacity to anticipate or experience pleasure; social anhedonia affects the ability to experience pleasure from interpersonal relationship. In schizophrenia, anhedonia can be a primary symptom of the illness, or a secondary symptom caused by depression. Although treatments often focused more on positive symptoms than negative ones, anhedonia has been shown to have a great impact on social functioning (Buchanan, 2007; Blanchard et al., 2011). It has become increasingly important to be able to recognize its presence and its cause in order to treat it.

Although anhedonia is mostly recognized as a key feature of the negative symptoms of schizophrenia, it is continuously distributed throughout the general population. Although there are several self-report measures of anhedonia, only the Revised Social Anhedonia Scale (RSAS) (Eckblad et al., 1982) and the Anticipatory and Consummatory Interpersonal Pleasure Scale (ACIPS) (Gooding et al., 2014) focus solely on social anhedonia. The 40-item RSAS is the most well-known measure of social anhedonia. Perhaps because the RSAS is focused primarily on detection of psychopathology, it consists of dichotomous items which have a low rate of endorsement in the general population.

Decreased capacity for social and interpersonal pleasure is also found in the non-clinical population at a lower rate (Fonseca-Pedrero et al., 2014); this leads some researchers to question whether anhedonia might be better conceptualized as a personality trait (Lincoln et al., 2016). The ACIPS is an indirect measure of social anhedonia that can be used to evaluate the presence of social anhedonia in patients as well as the general population. The ACIPS is a measure specifically designed to assess hedonic capacity for social and interpersonal pleasure. In contrast to the RSAS, the ACIPS is not focused on psychopathology, and its items have higher rates of endorsement. Indeed, a recent investigation (Gooding et al., 2017) indicated that the ACIPS and RSAS assess unique as well as overlapping aspects of social anhedonia. Relative advantages of the ACIPS include its brevity, ease of administration, and updated content. Other advantages of the ACIPS is that there are different versions (i.e., child, adolescent, adult) available, suitable to the age of the respondents.

To date, there is ample evidence of the construct validity of the ACIPS as an indirect measure of social anhedonia. Investigators found evidence of convergent validity in studies of non-clinical undergraduate students (Gooding and Pflum, 2014a,b; Gooding et al., 2014) and community participants (Gooding et al., 2015), where there were moderate and statistically significant positive correlations between social/interpersonal pleasure as measured by the ACIPS and measures of related constructs, such as anticipatory pleasure and consummatory pleasure, reward responsiveness, social connectedness, and sociability. They also observed moderate and inverse correlations between social/interpersonal pleasure (as measured by the ACIPS) and measures of social anhedonia (as measured by the RSAS), and the No Close Friends subscale of the Schizotypal Personality Questionnaire (SPQ) (Raine, 1991) and Schizotypal Personality Questionnaire-Brief (SPQ-BR) (Cohen et al., 2010). Evidence of discriminant validity was derived from its statistically non-significant associations with measures of unrelated constructs such as magical ideation, perceptual aberration, schizotypal ambivalence, and social desirability. Evidence of concurrent validity can be derived from a study of non-psychiatric adults (Kadison et al., 2015) in which social anhedonia showed a strong relationship with facial expressivity during the viewing of images of threatening stimuli and others in distress as well as a robust interaction with gender.

While the ACIPS was originally developed with the goal of distinguishing anticipatory and consummatory aspects of pleasure (i.e., a two-factor structure), subsequent analyses did not support that factorial structure. Rather, evidence has supported either a three- or four-factor structure (Gooding and Pflum, 2014a, 2016). Because the fourth factor was based on only one item in one investigation and two items in another, the four factor structure is therefore not adequately testable and may be unreliable.

One plausible hypothesis is that individuals suffering from schizophrenia might have deficits in anticipating pleasurable events more than consummatory pleasure (Mote et al., 2014). However, previous studies tend to show that the temporal aspects – the distinction between anticipation and consumption – are not evident in the ACIPS (Gooding and Pflum, 2016). Gooding, the primary developer of the ACIPS, asserts that the distinction between anticipation and consummation may be more difficult to disentangle in the case of social interactions, where even in the case of “in the moment” interactions, the experience of pleasure may reflect an amalgam of “in the moment,” historical, and anticipatory aspects of related interactions. Alternatively, Frost and Strauss (Frost and Strauss, 2016), assert that although the anticipatory items of the ACIPS do not raise concern, the consummatory items may lack construct validity. Consummatory ACIPS items do not rely on direct experimentation of feeling but rather on hypothetical circumstances. To measure the consummatory pleasure, the respondent would have to be in the situation provoking the feelings. Yet in the case of hypothetical reports like the ACIPS, the respondent would have to imagine himself in the situation where he would experience the particular feeling or emotion. This could partly explain why the anticipatory-consummatory structure is not relevant for the ACIPS.

To date, there have been two cross-cultural validations of the ACIPS. The factor structure and construct validity of the ACIPS has been investigated in Spanish and Chinese samples. In a non-clinical Spanish sample, the investigators (Gooding et al., 2016) observed that three factors (namely, Intimate social interactions, Social bonding in the context of media/communications, and Casual socialization) accounted for 79% of the variance. In addition, the second edition of the Beck Depression Inventory (BDI-II) (Beck et al., 1996) was administered concurrently with the ACIPS. The participants who reported a moderate or severe level of depressive symptoms had higher total ACIPS scores than participants who reported a minimal to mild level of depressive symptoms. Given that anhedonia is one of the symptoms considered in the depressive syndrome, these findings (Gooding et al., 2016) suggest that the ACIPS demonstrates concurrent validity. In sum, the results of the Spanish cross-validation study appears wholly consistent with the findings based on American samples.

The Chinese translation of the ACIPS (Chan et al., 2016) was administered to a non-clinical adult sample, along with translations of the SPQ and measures of general pleasure in order to assess convergent validity as well as its factor structure. The results of the factor analysis revealed a four factor solution: Friendship, Family and Intimacy-related relationships, General Social interactions, and Casual interactions/conversations. Similar to the American English and Spanish forms of the ACIPS, the Chinese translation of the ACIPS was characterized by high internal consistency (as measured by the ordinal alpha coefficient statistic). Consistent with findings based on American samples, the investigators also observed that the Chinese ACIPS scores were associated with scores on the No Close Friends and Constricted Affect subscales of the SPQ (Raine, 1991). Moreover, the ACIPS was moderately correlated with measures of consummatory and anticipatory pleasure.

The first goal of this study was to validate the structure of the French version of the adult ACIPS. The second objective was to verify whether a one, two or three factor solution is most appropriate for the ACIPS scale.

## Materials and Methods

### Participants

Participants were 263 volunteers who were enrolled in School of Nursing Sciences, La Source in Lausanne as pre-graduate students or professionals in continuous education courses (male 35% and female 65%, age 34.9 ± 11.7 years). Participants responded anonymously and voluntarily. They could not be identified and no personal data concerning their health were collected. Ethical approval was therefore not required for this study in accordance with national and institutional guidelines.

### Measure

The ACIPS Adult version (Gooding and Pflum, 2014a,b) is a 17-item self-report measure in which hedonic capacity for social and interpersonal engagement is rated on a 6-point Likert scale, ranging from 1 “very false for me” to 6 “very true for me.” Total scores on the ACIPS can range from 17 to 102. Lower total scores reflect greater likelihood of social anhedonia.

### Translation of the ACIPS

Translation of the measure was conducted in accordance with international guidelines for translation of psychological measures (Hambleton et al., 2005). The ACIPS was translated into French by JF and AN and back-translated by an independent translator in English. The back-translation was checked by the primary author of the scale (DCG) and adjustments were made to make the scale conceptually equivalent to the English version.

### Statistical Analyses

For the confirmatory factor analysis (CFA), Likert type items were treated as categorical ordinal and all the models were estimated using a robust weighted least squares estimator with adjustments for the mean and variance (WLSMV). This approach is more robust to non-normality than treating ordinal response scales as continuous. Three models were estimated. A one-factor model representing a general undifferentiated “pleasure” latent construct was first tested on the 17 ACIPS items. A two-factor model distinguishing anticipatory-pleasure (items 1, 3, 7, 8, 10, 14, and 15) and consummatory-pleasure (items 2, 4, 5, 6, 9, 11, 12, 13, 16, and 17) was tested next. A three-factor model including intimate social interactions (items 2, 3, 6, 7, 9, 10, 14, and 17), group social interactions (items 1, 4, 11, and 13) and social bonding, with the establishment also of connections (items 5, 8, 12, 15, and 16), was then tested. Finally, a bifactor model including one general factor and three orthogonal specific factors was estimated in order to model the unique influence of the specific factors above and beyond the effects of a general factor (that represented the commonality of all manifest variables). The general factor was defined on the basis of all items and the three specific factors were defined similarly to the three factor model. To identify the scale of the latent factors, one factor loading was fixed to one for each latent variable. Each model was compared to a more restrictive alternative including one or two fewer factors with a robust chi-square test using the DIFFTEST procedure. Because the bifactor model was not nested within other models and the AIC and BIC coefficients cannot be computed using WLSMV estimation, this model was compared to the others based on examination of indicators of goodness of fit. Several indicators of model fit were used: the root mean square error of approximation (RMSEA), the Comparison fit index (CFI) and the Tucker–Lewis fit index (TLI). Values of RMSEA ≤ 0.06 and CFI and TLI ≥ 0.95 are interpreted as good fit while values of RMSEA ≤ 0.08 and CFI/TLI ≥ 0.90 are often considered as indicating acceptable fit (Hu and Bentler, 1999). The reliability of the three subscales was estimated with McDonald’s model-based Omega (ω) coefficient (Canivez, 2016). All statistical analyses were performed with the Mplus statistical package version 7.4.

## Results

**FIGURE 1:**
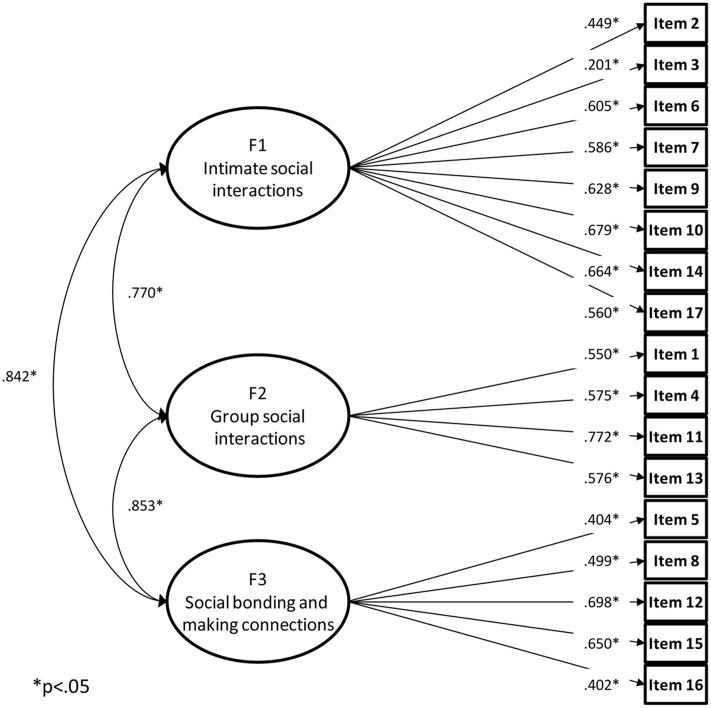
Three factor model for the ACIPS scale.

As shown in **Table 1**, the one and two-factor models showed somewhat poor fit to the data. However, the goodness of fit of the three factor model was acceptable. The results of the robust chi-square difference tests indicated that while the two-factor model did not improve on the one-factor model fit (2 factors against 1 factor: Δχ^2^ = 1.638, Δ*df* = 1, *p* = 0.201) the 3 factor solution should be preferred overall (3 factors against 1 factor: Δχ^2^ = 23.218, Δ*df* = 3, *p* < 0.001; 3 factors against 2 factors: Δχ^2^ = 26.337, Δ*df* = 2, *p* < 0.001). This model is represented in **Figure 1**. This general factor accounted for 70.1% of the common variance (intimate social interactions 14.3%, group social interactions factor 6.6% and social bonding and making connections factor = 8.6%).

**Table 1:** Comparisons of Model fit for the ACIPS scale.

Model	χ^2^	*df*	*p*-value	RMSEA	90% C.I for RMSEA	CFI	TLI
One factor model	235.266	119	<0.001	0.061	0.049–0.072	0.895	0.880
Two factor model	234.198	118	<0.001	0.061	0.050–0.073	0.895	0.879
Three factor model	216.692	116	<0.001	0.057	0.045–0.069	0.909	0.894
Bifactor model	188.643	102	<0.001	0.057	0.044–0.069	0.922	0.896

*df, degree of freedom; RMSEA, Root Mean Square Error of Approximation; CI, Confidence Interval; CFI, Comparative Fit Index; TLI, Tucker-Lewis Index*.

All factor loadings were statistically significant and the three factors correlated substantially with each other. The reliability of the three scales (ω intimate social interactions = 0.789, ω group social interactions = 0.726, ω social bonding and making connections = 0.700) was satisfactory to good (Canivez, 2016). While being the less parsimonious model because it included four factors, the model fit of the bifactor model was very similar to the three factor model. All loadings on the general factor were supported while the loadings of items 3, 4, 5, 6, 10, 12, and 13 on their specific factors were no longer statistically significant.

## Discussion

The first aim of this study was to confirm the factor structure of the French version of the ACIPS. The psychometric characteristics are similar to the original version (Gooding and Pflum, 2014b). Our findings are thus consistent with previous studies on the English and Spanish version (Gooding and Pflum, 2016; Gooding et al., 2016).

Our second goal was to determine which factor solution was the best fit to the ACIPS data. The one and two-factor – anticipatory-consummatory pleasure – models did not show good fit to the data, which also confirms previous findings (Gooding and Pflum, 2016). Some respondents experience difficulties in imagining themselves in a hypothetical situation (Frost and Strauss, 2016), which would explain the lack of difference between anticipatory and consummatory pleasure. The model including “intimate social interactions,” “group social interactions,” and “social bonding and making connections,” shows that the three factors correlated well with each other. All items contributed to their corresponding factors and showed adequate model based reliability. This structure seems consequently to be the most adequate for the ACIPS. The “intimate social interactions,” “group social interactions,” and “social bonding and making connections” factors were substantially correlated and shared a large amount of variance. These results suggest that individuals who enjoyed interaction in one of these three subdomains were more likely to enjoy doing so in the two other domains.

While on the basis of the comparison between the one and three factor models, these three types of interactions may not be considered as indistinguishable and to represent distinct and theoretically meaningful dimensions, results of the bifactor model highlighted that all items also measured general undifferentiated “pleasure.” This suggests that some items did not measure distinct specific pleasure beyond and above general undifferentiated pleasure. Taken together, these results show the ACIPS is a valid scale to measure the capacity of savoring different kinds of social relationships.

The item with the lowest corresponding coefficient to its factor is “I’m not looking forward to seeing my family” (item 3; see **Figure 1**), which is the only negatively worded item. It is often seen that respondents take some time to understand that item, or understand it the wrong way because of its unique sentence structure. It might be interesting to reword the item in a positive way for further studies. Another future direction for the ACIPS as a self-report scale would be to include patients in the identification of their interpersonal pleasure deficits and use the scale to help personalize interventions. It should be noted that despite the absence of relevance of the two-factor model for the ACIPS, therapies which focus on anticipating pleasurable moments are likely to be beneficial to patients with demonstrable deficits in these areas, such as patients with schizophrenia (Edwards et al., 2015). Self-report scales may be limited because we tend to think about our reaction to the most recent event or they tend to be decontextualized. Despite the limitations inherent in self-report measures, the ACIPS can be very useful in clinical as well as in non-clinical settings, where social anhedonia is also to be found (Brown et al., 2007). The ACIPS can be used to identify and follow the course of social anhedonia, which appears as a transdiagnostic symptom in several different disorders (Bedwell et al., 2014). Alternatively, the ACIPS can be used as an outcome measure in terms of investigating response to experimental treatments. Our results suggest further psychological intervention could focus on these three different levels of social bonding.

Limitations of the present study could be addressed in future research. The disproportionate number of females in the present sample (i.e., two-thirds female) may limit the generalizability of our findings. Replication with a larger proportion of male participants is therefore advisable. The results presented are based entirely upon non-clinical samples of high functioning individuals recruited through university and continuous post-grade education. It would be interesting to compare these findings with those derived from clinical populations, such as patients with schizophrenia. Moreover, the current study design is cross-sectional. Given the temporal stability of the measure (Gooding and Pflum, 2016), the ACIPS is suitable for treatment evaluation studies. It would be useful to examine the ACIPS scores of individuals before and after interventions aimed at reducing anhedonia (Favrod et al., 2015; Nguyen et al., 2016).

The present study demonstrated the internal consistency of the ACIPS scale in French-speaking non-clinical individuals. However, this line of research would be furthered by inclusion of measures of other theoretically related and unrelated constructs in order to measure the convergent and discriminant validity of the ACIPS, respectively.

This study also provided the first evidence of cross-cultural validity of the ACIPS in a French-speaking context and supported the use of the scale in cross-cultural research. That is, the results of the present investigation indicate that the adult version of the ACIPS was successfully adapted from American English to French with semantic, linguistic, and contextual equivalence. Such psychometric evidence is an important step in the process of demonstrating structural invariance (i.e., relationship between factors) of the ACIPS. Taking into account data from Chinese, Spanish, American English and the present investigations, one can assert that the ACIPS has demonstrated structural variance, though questions regarding measurement invariance (i.e., relationship between items and factors) remain. Future directions could include the use of multidimensional scaling in order to assess the comparability of the ACIPS in different populations and cultures (Reise et al., 1993; Brown, 2006; Borsa et al., 2012).

## Author Contributions

JF, PG, and AN designed this research. DG conceptualized and developed the scale. JF, AN, and CF acquired the data. PG analyzed and interpreted the data. JC, PG, and JF drafted the first version of the manuscript. DG, AN, and CF critically revised the article for important intellectual content. All the authors approved the final version for publication. All the authors agree to be accountable for all aspects of the work by ensuring that any questions related to its accuracy or integrity can be appropriately investigated and resolved.

## Conflict of Interest Statement

The authors declare that the research was conducted in the absence of any commercial or financial relationships that could be construed as a potential conflict of interest.
